# *Megasphaera elsdenii* Lactate Degradation Pattern Shifts in Rumen Acidosis Models

**DOI:** 10.3389/fmicb.2019.00162

**Published:** 2019-02-07

**Authors:** Lianmin Chen, Yizhao Shen, Chao Wang, Luoyang Ding, Fangfang Zhao, Mengzhi Wang, Jingyuan Fu, Hongrong Wang

**Affiliations:** ^1^Laboratory of Metabolic Manipulation of Herbivorous Animal Nutrition, College of Animal Science and Technology, Yangzhou University, Yangzhou, China; ^2^Department of Pediatrics, University Medical Center Groningen, University of Groningen, Groningen, Netherlands; ^3^Department of Genetics, University Medical Center Groningen, University of Groningen, Groningen, Netherlands; ^4^School of Biomedical Sciences, The University of Western Australia, Crawley, WA, Australia; ^5^School of Animal Biology, The University of Western Australia, Crawley, WA, Australia

**Keywords:** rumen acidosis, rumen microbiota, starch fermentation, lactate, lipopolysaccharide

## Abstract

**Background:** *Megasphaera elsdenii* is an ecologically important rumen bacterium that metabolizes lactate and relieves rumen acidosis (RA) induced by a high-grain-diet. Understanding the regulatory mechanisms of the lactate metabolism of this species in RA conditions might contribute to developing dietary strategies to alleviate RA.

**Methods:** *Megasphaera elsdenii* was co-cultured with four lactate producers (*Streptococcus bovis*, *Lactobacilli fermentum*, *Butyrivibrio fibrisolvens*, and *Selenomonas ruminantium*) and a series of substrate starch doses (1, 3, and 9 g/L) were used to induce one normal and two RA models (subacute rumen acidosis, SARA and acute rumen acidosis, ARA) under batch conditions. The associations between bacterial competition and the shift of organic acids’ (OA) accumulation patterns in both statics and dynamics manners were investigated in RA models. Furthermore, we examined the effects of substrate lactate concentration and pH on *Megasphaera elsdenii’s* lactate degradation pattern and genes related to the lactate utilizing pathways in the continuous culture.

**Results and Conclusion:** The positive growth of *M. elsdenii* and *B. fibrisolvens* caused OA accumulation in the SARA model to shift from lactate to butyrate and resulted in pH recovery. Furthermore, both the quantities of substrate lactate and pH had remarkable effects on *M. elsdenii* lactate utilization due to the transcriptional regulation of metabolic genes, and the lactate utilization in *M. elsdenii* was more sensitive to pH changes than to the substrate lactate level. In addition, compared with associations based on statics data, associations discovered from dynamics data showed greater significance and gave additional explanations regarding the relationships between bacterial competition and OA accumulation.

## Introduction

Current intensive livestock management systems encourage the inclusion of large amounts of cereal grains in the diets of ruminants to support a high production rate and enhance cost efficiency. After feeding ruminants with high grain diets, non-fibrous carbohydrates (NFC, mainly starch) will arrive at the rumen, thereby promoting the fermentation of amylolytic bacteria, e.g., *Streptococcus bovis* and *Lactobacilli* spp. to produce pyruvate and finally converting to organic acids (OA) (Wang et al., 2015; Mickdam et al., 2016). In this process, *S. bovis*, which is the most prominent amylolytic bacterium in the rumen, proliferates rapidly and primarily produces lactate while limit amount of volatile fatty acids (VFA) (Russell and Hino, 1985; Asanuma and Hino, 2000; Hernández et al., 2014; Chen et al., 2016a,b,c; Chen and Wang, 2016). During the early period, an insignificant amount of lactate is quickly metabolized by lactate utilizers, especially *Megasphaera elsdenii*, and is converted into acetate, propionate, and butyrate (Chen et al., 2016c; Chen and Wang, 2016). However, once asynchronous fermentation occurs between lactate-producing and lactate-utilizing bacteria, the lactate accumulates in the rumen, the pH of the ruminal fluid drops rapidly (Hernández et al., 2014; Kenney et al., 2015; Wang et al., 2015), and rumen acidosis (RA) is induced. In general, if the rumen’s pH declines below 6, it is recognized as subacute rumen acidosis (SARA) (Therion et al., 1982; Asanuma and Hino, 2001), whereas a ruminal pH below 5.0 is defined as acute rumen acidosis (ARA) (Giesecke and Stangassinger, 1980; Hernández et al., 2014). Owing to low pH in both ARA and SARA, most gram-negative bacteria undergoes lysis, resulting in the release of endotoxin, e.g., lipopolysaccharide (LPS) into the ruminal fluid, which can trigger systemic inflammation (Chin et al., 2006; Nagaraja and Titgemeyer, 2007; Loor et al., 2016).

As an important lactate utilizer, the administration of *M. elsdenii* has been reported as ameliorating rumen fermentation and relieving RA (Long et al., 2014; Weimer et al., 2015), though with different potency. In addition to the difference in strain diversity and doses (Long et al., 2014, 2016; Meissner et al., 2014; Muya et al., 2015; Weimer et al., 2015; Leeuw et al., 2016), the complexity of competition amongst rumen microbiota and/or their interaction, as well as the fermentation environment, i.e., substrate abundance and pH, might have effects on *M. elsdenii* lactate degradation. Although some studies have already focused on the lactate degradation pathway of *M. elsdenii* (Prabhu et al., 2012; Weimer and Moen, 2013), little is known about lactate utilization by *M. elsdenii* under RA conditions. Understanding the regulation of lactate metabolism in this species might contribute to developing dietary strategies to alleviate RA.

Previous work by our group and others has indicated that several bacterial populations (*S. bovis*, *Lactobacilli* spp., *M. elsdenii*, *Butyrivibrio fibrisolvens*, and *Selenomonas ruminantium*) associated with lactate metabolism play significant roles in the initiation of RA (Khafipour et al., 2009; Mao et al., 2013; Wang et al., 2015). Given that these bacteria have already been isolated, it is possible to study the lactate degradation of *M. elsdenii* in a mixed culture of RA models. Therefore, we used different starch concentrations to induce RA models in co-culture, and aimed to understand how *M. elsdenii* compete (interact) with lactate producers to metabolize lactate in both statics and dynamics manners. In addition, we examined the effects of lactate concentrations and pH on *M. elsdenii* lactate degradation and the expression of genes involved in lactate utilization in continuous culture.

## Experimental Procedures

### Strains and Seed Culture

Four lactate producers *S. bovis* (CCTCC AB2016240, produces lactate mainly and also formate and acetate) (Chen et al., 2016b), *L. fermentum* (ATCC 11976, produces lactate), *B. fibrisolvens* (ATCC 27208, produces lactate, formate, and acetate) (Bryant and Small, 1956), *S. ruminantium* (ATCC 12561, lactate mainly and also formate, acetate, and butyrate) (Bryant, 1956) and one lactate utilizer *M. elsdenii* (ATCC 25940, can produce acetate, propionate, and butyrate) (Elsden et al., 1956) were used in this study. All strains were cultured in a non-rumen fluid media (Caldwell and Bryant, 1966) with 2% (vol/vol) fetal bovine serum (PN.10082147, Gibco, Langley, OK, United States). All media (except fetal bovine serum) were sterilized by autoclaving at 121°C and 15 psig for 15 min. The culture was grown in an anaerobic workstation (DG250, Don Whitley Scientific Limited, Bingley, England) with 10% H_2_, 10% CO_2_, and 80% N_2_ (mol/mol) at 37°C.

### Batch Culture and Sampling

Unless otherwise stated, the bacteria were grown in 250 mL serum bottles containing 200 mL of media at 37°C in an anaerobic shaking water bath (37°C, 160 rev/min). The modified basal medium (Russell and Baldwin, 1979) for mixed culture contained the following in 1 L: 1.0 g of tryptone, 0.5 g of yeast extract, 0.6 g of cysteine-HCl, 4.0 g of Na_2_CO_3_, 0.5 g of (NH_4_)_2_SO_4_, 0.4 g of NaHCO_3_, 8.0 mg of CaCl2H_2_O, 8.0 mg of MgSO_4_7H_2_O, 40.0 mg of K_2_HPO_4_, 40.0 mg of KH_2_PO_4_, 80.0 mg of NaCl, 5.40 mL of acetic acids, 1.86 mL of propionic acids, 1.23 mL of butyric acids, 0.10 mL of isobutyric acid, and 0.10 mL of valeric acids. The experimental treatments included three levels of soluble starch (PN. S9765, Sigma, United States) as the sole carbohydrate source included in the media: 1 g (limit level), 3 g (close to a normal range in the rumen fluid), and 9 g (excessive level). The pH of the media was maintained at 7.0 using 10% NaOH (wt/vol). When the seed culture of each strain reached an optical density (OD) of 0.3 (exponential phase), the cells were collected by centrifugation at 2000 rpm for 4 min at 4°C, and the strains were mixed as follows: 10% *S. bovis*, 15% *L. fermentum*, 25% *B. fibrisolvens*, 10% *M. elsdenii*, and 40% *S. ruminantium*. The mixing ratio (No./No.) of each strain was based on the rumen bacterial composition under normal conditions found in our previous study of dairy cows (Wang et al., 2015). For the culture, 1.0 mL (2 × 10^8^ CFU/mL) of the mixed seed culture was used as the inoculator. All treatments were in quadruplicate. Cell growth was estimated by measuring the OD values at 600 nm. The change of media pH was monitored using a pH meter (Seven Excellence equipped with InLab Routine Pro-ISM meter, Mettler-Toledo, Switzerland). Samples of fermentation fluid (3 mL each) were drawn from the bottles using syringes at 0, 3.5, 8.5, 11.5, 14.5, and 22.5 h for the measurements listed below.

### Continuous Culture and Sampling

The basal medium for continuous culture contained the following in 1 L: 1.0 g of tryptone, 1.0 g of yeast extract, 0.6 g of cysteine-HCl, 0.9 g of K_2_HPO_4_, 0.9 g of KH_2_PO_4_, 1.8 g of NaCl, 0.24 g of CaCl_2_⋅2H_2_O, 0.83 g of MgSO_4_⋅7H_2_O, and 1.8 g of (NH_3_)_2_SO_4_ (Hino and Kuroda, 1993). The experimental treatments were of a 3 × 2 factorial design: three levels of DL-lactate (Sigma, PN. 69785) as the sole carbohydrate source in the media (Counotte et al., 1981; Henning et al., 2010): 15 mM (close to a normal range in the rumen fluid), 30 mM (close to a range of SARA in the rumen fluid), and 90 mM (excessive level), respectively, while the pH of the media was maintained constantly at 5.5 (acidosis) or 6.5(normal) using 10% NaOH (wt/vol) or 10% HCl (vol/ vol). Quadruplicates were used for each treatment. For the culture, 2.0 mL of the *M. elsdenii* culture was used as the inoculator once the culture had attained the OD of 0.4 (exponential phase). Cell growth was monitored by measuring OD values at 600 nm using a SpectraMax M5 plate reader (Molecular Devices, Silicon Valley, CA, United States). The medium fluid was, respectively, collected at 4.5 h (during the lag phase), 7 h (during the exponential phase), and 24 h (during the plateau phase) for analysis of the fermentation products, bacterial enumeration, and gene expression.

### Determination of Total Amount of Sugar, Enzyme Activity and Organic Acids Concentration

Bacterial cell homogenization was performed by mixing 2 mL of bacterial fluid with 0.3 g of zirconia-silica beads (0.1 mm in diameter). The samples were then homogenized in a FastPrep-24 Automated system (MP Biomedicals, Solon, OH, United States) for 30 s, followed by sonication for 1 min (100 W, 30 cycles) using a VCX-130 sonicator (Sonics, United States) in an ice bath. Cell debris was removed by centrifugation at 5000 rpm for 8 min at 4°C, and the supernatant was collected for further analysis.

Total sugar in the media was assayed using a phenol-sulfuric acid method (Nielsen, 2010) using a MAK104 kit (Sigma, United States) according to the manufacturer’s protocol.

The OA (lactate, formate, acetate, propionate, and butyrate) concentrations in the supernatant were measured using a high-performance liquid chromatography (HPLC, Shimadzu SPD-15C, Japan) equipped with an acclaim OA column (PN. 062902, Thermo Fisher Scientific, Waltham, MA, United States) and a UV detector. The column temperature was maintained at 30°C, the mobile phase was 100 mM Na_2_SO_4_ (pH 2.65, adjusted with methanesulfonic acid), and the flow rate was set to 0.6 mL/min. OA were then measured at UV 210 nm.

The activities of the intracellular enzymes lactate dehydrogenase (LDH) and α-amylase (α-AMY) in the supernatant were measured as follows: LDH was measured using a method similar to that of De Vries et al. (1970) with a commercial kit (PN. A020, Jiancheng Biotech Co., Ltd., China), and α-Amy activity was measured using an iodine–starch method (Gil’manov et al., 1981) with a C016-1 kit (Jiancheng Biotech Co., Ltd., China). The analyses were carried out according to the manufacturer’s instructions.

### LPS Assay

After sampling, 1 mL of bacterial fluid was centrifuged immediately at 4000 rpm for 5 min at 25°C, and the supernatant was collected and concentrated (1/10 times, Concentrator plus, Eppendorf, Germany) for LPS analysis, while the cells were collected for genomic DNA extraction. LPS was measured with an enzyme-linked immunosorbent assay kit (PN. CEB526Ge, Cloud-Clone Corp., United States) following the manufacturer’s directions.

### Bacterial Quantitation and Gene Transcription

For bacterial quantitation, the qPCR technique was used, whereas for gene expression, RT-qPCR was used. Bacterial cells were stored in 2 mL RNAlater reagent (Qiagen, Germany). During isolation, 200 μl of lysozyme solution (10 mg/mL) was added to collected bacteria and incubated at 37°C for 5 min to break down the cell walls. Genomic DNA was then extracted using a QIAamp DNA Stool Mini Kit (Qiagen, Germany). Total RNA was extracted using a RNeasy Midi Kit (Qiagen, Germany). Genomic DNA and total RNA quality was assessed using NanoDrop 1000 (NanoDrop Technologies LLC, Wilmington, DE, United States) with 260/280 around 1.75 and 1.9, respectively. The RNA was reverse-transcribed to cDNA with random-hexamer primers and Omniscript RT Kit (Qiagen, Germany). qPCR was performed in an ABI Real-Time PCR (ABI 7500, United States), operated according to the manufacturer’s instructions (204054, Qiagen, Germany). The reaction was first performed in a 20-μL reaction solution containing 10 μL of 2× QuantiFast SYBR Green PCR Master Mix, 1.6 μL of primer, 1 μL of DNA template (100 ng) or cDNA template (300 ng), 0.4 μL of ROX and 7.0 μL of RNase-free water. Following a single pre-denaturation cycle at 95°C for 5 min, the amplification was performed for 45 cycles (95°C for 10 s and 60°C for 32 s). The 16*S* (AbuGhazaleh et al., 2011) gene was used as reference gene, and primers for other genes involved in *M. elsdenii* lactate degradation are shown in Supplementary Tables S1, S2, respectively. PCR products were electrophoresed on a 1% agarose gel and visualized upon staining with ethidium bromide to detect the specificity of the polymerase chain reaction (Supplementary Figures S1, S2). Gene expression was calculated according to (Pfaffl, 2001), and PCR amplification efficiency was calculated according to (Ramakers et al., 2003). The relative expression quantity = $\frac{\left(\text{Et})\;\Delta \text{CPt }(\text{control}-\text{sample}\right)}{\left(\text{Er})\;\Delta \text{CPr }(\text{control}-\text{sample}\right)}$, where *E_t_* is the real-time PCR efficiency of target gene transcription, E_*r*_ is the real-time PCR efficiency of a reference gene, ΔCPt is the CP deviation of control – sample of the target gene, and ΔCPr is the CP deviation of control – sample of reference gene.

### Statistical Analysis

The results were expressed as the least square mean ± standard error of means (SEM), and statistical analyses were performed by using R statistical software (version 3.4.3, The R Foundation^1^). In co-culture models, principal component analysis (PCA) was used to distinguish RA models. The two-way ANOVA model for repeated measures was used to examine the fixed effects of time (six time points), soluble starch concentrations (three levels) and their interactions. The multiple comparisons were performed using Tukey’s test. In order to attend to the differences among the RA models, multiple comparisons were performed on the means of the starch concentrations across all six sampling times, as well as the observed value at each time point for all three starch concentrations in three models. Relative growth (production) rate was calculated using the following model: k_*t*_ = (A_*t*_+Δt – A_*t*_−Δt) /2Δt, where k_*t*_ is the relative growth (production) rate at time t, A_*t*_ is the relative abundance (amount) of bacteria (OA) at time t, Δt is average time interval of determination. A multiple linear regression (MLR) model: Y = a + b_1_X_1_ + b_2_X_2_ + … +b_*i*_X_*i*_ (Y is the dependent variable, a is the constant term, X_*i*_ is the independent variables, b_*i*_ is the regression coefficients) was used based on Z score (Z = (x − μ)/σ, where μ is the mean of all the measures, σ is the standard deviation of all the measures) to evaluate the associations between bacteria growth and OA production both in statics and dynamics. In continuous culture models, linear regression was conducted to analyze the growth rate of the *M. elsdenii* during the log phase. The model is as follows: Y = kX + b, where Y is the OD reading, X is the hour of the incubation (h), k is the steepness of the curve (i.e., growth rate, OD units per hour), and b is the constant term. The three-way ANOVA model for repeated measures was used to examine the fixed effects of time (three time points), substrate lactate concentrations (three levels), pH (two levels), and their interactions. The multiple comparisons were also performed using Tukey’s test. An MLR based on the Z score was used to evaluate the associations between *M. elsdenii* growth and OA production. *P* values of less than 0.05 (*P* < 0.05) were considered to be significant.

## Results

### Characterizing RA Models

**FIGURE 1:**
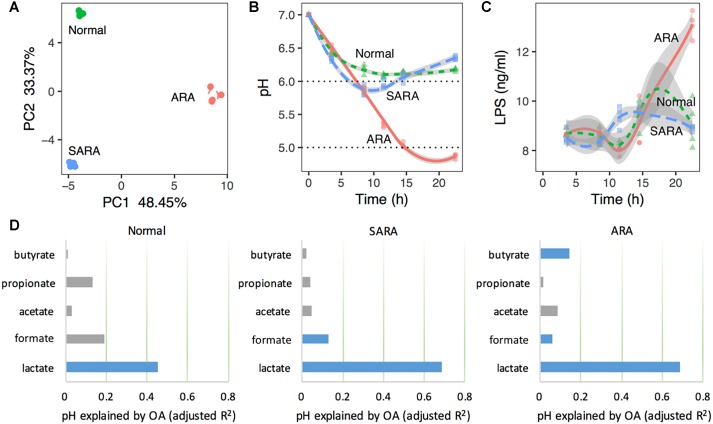
Rumen acidosis models (RAMs) can be induced by substrate starch abundance in mixed culture. **(A)** Principal component analysis (PCA) based on pH, bacteria relative abundance, organic acids concentration, LPS, and enzymes’ activity. **(B)** pH shifts in RAMS. **(C)** LPS concentrations at different sampling time points in RAMs. **(D)** Variation of pH explained by organic acids’ concentration in RAMs with model: pH ∼ lactate + formate + acetate + propionate + butyrate. Blue bars are statistically significant (*P* < 0.05). The *x*-axis represents the variance explained by organic acids. The total variance is 1.

In order to classify rumen conditions, PCA was used based on the datasets of pH, bacteria relative abundance, OA concentration, LPS, and enzymes’ activity. According to the first and second principal components (Figure 1A), there were three separated clusters, suggesting that the three models were distinct. Moreover, in each model, samples were tightly clustered (Figure 1A), reflecting excellent reproducibility. It was noteworthy that the samples were clustered based on the abundance of substrate starch in the media, suggesting that the starch levels provided the driving force between different clusters.

Acute rumen acidosis is characterized by a dramatic reduction in the ruminal pH below 5.0, and SARA is defined as periods of moderately depressed ruminal pH lower than 5.8 for 4 h or more after feeding, while the rumen pH should normally be above 5.8 (Nagaraja and Titgemeyer, 2007; Loor et al., 2016). Here, substrate starch levels had significant effects on media pH (*P* < 0.001, Figure 1B and Supplementary Table S3). We further characterized the media pH of each cluster and named three RA models. Briefly, a normal model was characterized by a slight drop of pH with time and finally became stable (from 11.5 h), but nevertheless remained above 6.0 (Figure 1B); a SARA model showed that pH decreased first (reaching the lowest pH of 5.8 at 8.5 h) and then recovered (reaching a final pH of 6.4 at 22.5 h), with a pH range from 5.8 to 5.9 for a duration of 5 h (Figure 1B); a ARA model showed that pH declined quickly with time, reaching a final pH of 4.7 (below 5.0) at 22.5 h (Figure 1B). We found that the starch concentrations could very effectively represent different RA models. These results suggest that the present *in vitro* RA models showed similarity with *in vivo* RA models in terms of pH changes upon a high-starch diet.

In order to ascertain the importance of lactate to pH changes in RA models, MLR was used based on the Z score, and the results proved that lactate played a dominant role in regulating pH (Figure 1D, explained more than 40% variance in normal, *P* = 7 × 10^−5^, and more than 60% variance in two RA models, *P* < 10^−8^). This indicated that the changes in pH in different models can be driven by lactate.

Aside from pH, the LPS concentration in the rumen fluid was also important in reflecting RA. Here, the average concentration of LPS in ARA was significantly higher than that of normal and SARA (*P* < 0.001, Supplementary Table S3), but no significant difference between normal and SARA was found in average concentration (*P* = 0.659, Supplementary Table S3). Moreover, except at 11.5 and 22.5 h, there were no significant differences in LPS concentration among the three RA models (*P* > 0.05, Figure 1C).

### Organic Acids Accumulation Associated With Bacterial Abundance in Statics Manners

In order to ascertain a better understanding of the differences among the models, we calculated the proportion of each species (Supplementary Figure S3A) and OA (Supplementary Figure S3B) in the models across five sampling time points. With the progress of fermentation, the proportion of *S. bovis* overall increased (especially in the normal model) whereas *S. ruminantium* overall declined (especially in ARA) in all three of the models (Supplementary Figure S3A). The percentage of *M. elsdenii* overall declined in normal and ARA models, but overall increased in SARA (especially from 3.5 to 8.5 h, Supplementary Figure S3A). However, the proportion of *B. fibrisolvens* overall declined in normal and SARA, but dropped to 19% at 11.5 h and finally increased to 33% in ARA. Except the changes in species composition, the composition of OA also changed considerably. Here, the proportion of lactate overall increased with time in ARA, and ultimately reached 50% (Supplementary Figure S3B). However, in SARA, lactate reached a proportion of 30% at 8.5 h, before dropping to 2% at 22.5 h (Supplementary Figure S3B). When considering shifts in the proportion of butyrate, we found that it overall declined with time in normal and ARA (10 and 6%, respectively, at 22.5 h), but quickly increased in SARA from 11.5 (17.5%) to 22.5 h (31%) (Supplementary Figure S3B). The percentages of acetate and formate were higher in normal and SARA than in ARA, while the proportion of propionate in RA models were lower than normal (Supplementary Figure S3B). These results reflect the differences and complexities of bacterial competition in joint fermentation and finally leading to the shifts of OA pattern.

**FIGURE 2:**
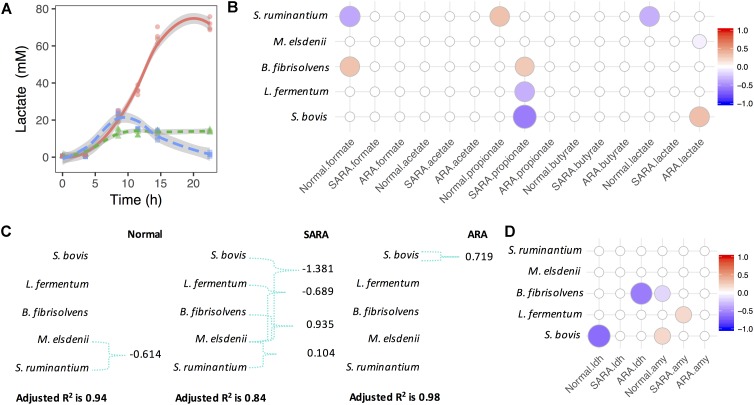
*Megasphaera elsdenii* combats lactate producers to maintain lactate balance in RAMs. **(A)** Lactate accumulation in RAMs. **(B)** Associations between bacteria relative abundance and OA accumulation in RAMs. Only significant associations were presented (*P* < 0.05). **(C)** The variation of lactate concentration explained by bacteria abundance and interactions. Green connections are statistical significant interactions (*P* < 0.05) and the model used here is: lactate ∼ sb + me + bf + lf + sr + me:(sb + bf + lf + sr). **(D)** Associations between bacteria relative abundance and enzymes’ activity in RAMs. Only significant associations were presented (*P* < 0.05).

In order to investigate the association between bacteria competition and OA accumulation, MLR analysis was used based on the Z score of statics-observed values in each model. In the normal model, we saw significant growth of *S. bovis* and increase of lactate and formate concentrations, but no significant association between *S. bovis* and OA was discovered (Supplementary Figure S4 and Figure 2B). However, the lactate producer *S. ruminantium* was significantly associated with formate and lactate (negative association, *P* < 0.003), and positively associated with propionate (*P* = 0.04), although changes in propionate were limited (Figure 2B and Supplementary Figure S4). In SARA, aside from the rapid growth of *S. bovis*, *M. elsdenii*, and *B. fibrisolvens* also increased (Supplementary Figure S4). Interestingly, the lactate level in SARA increased to 24 mM at 8.5 h and then quickly declined to 2 mM at 22.5 h (Figure 2A). During this period, butyrate increased considerably (from 10 to 24 mM, Supplementary Figure S4) and lactate utilizer *M. elsdenii* also increased (1.5 times, Supplementary Figure S4), but no significant association was seen among bacteria abundance, lactate, and butyrate concentrations (Figure 2B). Given that the present MLR model only considered the independent effect of bacteria, it was unable to reflect the relationships between bacteria and lactate utilization.

For further exploration of lactate production and utilization in bacteria competition, we considered lactate producer-lactate utilizer interactions (Figure 2C). We found that in the normal model, the variation of lactate concentration can only be significantly explained by the interaction between *S. ruminantium* and *M. elsdenii* (*P* = 0.006), while in the SARA model, the variation of lactate concentration was significantly explained by interactions between *M. elsdenii* and lactate producers (*P* < 0.01, Figure 2C). In ARA, only a significant positive association between *S. bovis* and lactate concentration (*P* = 0.001, Figure 2C) was found, while in the “without interaction” model, a significant negative association was seen between *M. elsdenii* and lactate concentration (*P* = 0.018, Figure 2B).

We also found several associations between the abundance of lactate producers and the activity of lactate synthesis related enzymes (Figure 2D and Supplementary Figure S5). Suggesting that lactate producers might play important roles in lactate joint fermentation. There were also significant negative associations between the abundance of lactate producers (i.e., *S. bovis* and *L. fermentum*) and propionate levels in SARA (*P* < 0.007, Figure 2B), whereas *B. fibrisolvens* acted in a positive way (*P* = 0.01, Figure 2B). These observations highlight the different roles played by bacteria in terms of OA joint fermentation in statics manner, especially in lactate production and utilization.

### Organic Acids Concentrations Associated With Bacteria Abundance in Dynamics Manners

It is possible to obtain large amounts of information regarding bacteria competition and its effects in OA joint fermentation based on statics data. However, these static snapshots of bacteria and fermentation at the point of collection cannot be used to represent the highly dynamic nature of bacteria. Instead, bacteria competition should be more dynamic, rather than static. Here, we calculated the mean growth/production rates of bacteria and OA, and an MLR based on the Z score was used to investigate associations between OA production rate and bacteria growth rate.

**FIGURE 3:**
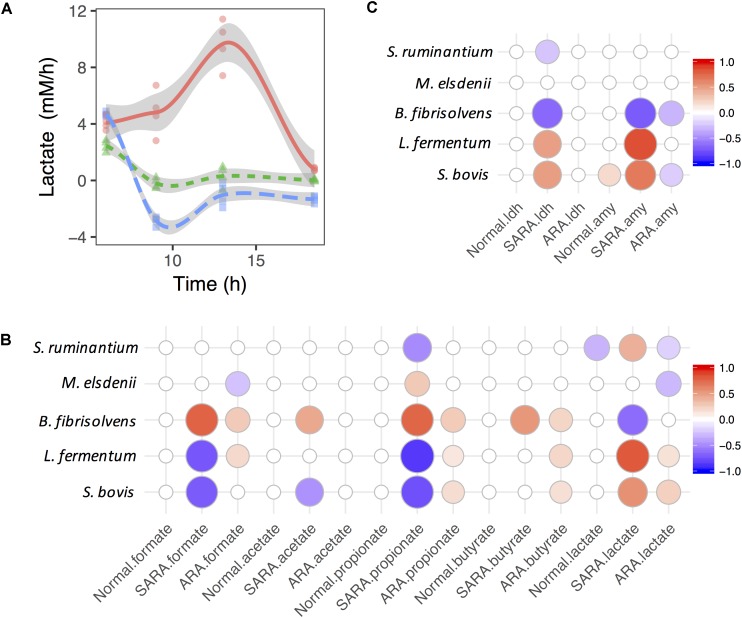
Associations between bacteria growth and OA accumulation in dynamics. **(A)** Shift of lactate accumulation rate in RAMs. **(B,C)** are associations between bacteria growth rates and accumulation rates of OA and enzymes. Only significant associations were presented (*P* < 0.05).

The production rate of lactate in the ARA model increased first before decreasing, but it showed the opposite direction in the normal and SARA models (Figure 3A). In the normal model, the growth rate of *S. bovis* increased first and then declined (remaining above 0), while the production rate of lactate dropped slightly and finally fell below 0 (Supplementary Figure S6). The *S. bovis* also had a significant positive association with α-AMY activity’s increase rate (*P* = 0.02, Figure 3C), but only the lactate producer *S. ruminantium* played a significant negative role in lactate production dynamics in the normal model (*P* = 0.006, Figure 3B). This suggests that lactate production/utilization competition continued to exist even though all of the bacteria except for *S. bovis* were disappeared.

In the SARA model, the lactate production rate quickly dropped below 0 (Supplementary Figure S6), suggesting that the lactate production rate was less than the utilization rate. In contrast to the lactate production rate, the butyrate production rate increased quickly and then declined (Supplementary Figure S6), but no significant association was found between the lactate utilizer and butyrate in dynamics (*P* > 0.05, Figure 3B). During this period, the growth rate of *S. bovis*, *B. fibrisolvens*, and *M. elsdenii* all showed a similar trend (increase then drop, Supplementary Figure S6). We also found three lactate producers (*S. bovis*, *S. ruminantium*, and *L. fermentum*) showed positive association with the lactate production rate but *B. fibrisolvens* showed opposite direction (Figure 3B). The same association directions were also found between lactate producers’ growth rates and enzymes’ activity increase rates (Figure 3C).

When considering ARA, lactate producers (especially *S. bovis*) were positively associated whereas utilizers (especially *M. elsdenii*) were negatively associated with the lactate production rate (Figure 3B). However, only significant negative associations were discovered between lactate producers (*S. bovis* and *B. fibrisolvens*) and the α-AMY activity increase rate (Figure 3C). Moreover, lactate producers also had significant positive associations with formate, propionate, and butyrate production rates, suggesting the diversification and synergy of lactate producers’ OA joint fermentation in ARA.

### Substrate pH and Lactate Level Can Regulate *M. elsdenii* Lactate Degradation Pathway in Continuous Culture

Aside from the competition and interaction that affect *M. elsdenii* lactate degradation in mixed culture RA models, pH, and substrate abundance shifts may also play a remarkable role in regulating *M. elsdenii* lactate degradation pathways, as the present co-culture models were based on batch culture but not steady state conditions. In order to further investigate *M. elsdenii* lactate degradation under the RA pH and substrate lactate level, a continuous culture was carried out with a pH controlled at 6.5 (normal ruminal pH) or 5.5 (ruminal pH under RA) and substrate lactate doses set at 15 mM (close to a normal range in the rumen fluid), 30 mM (close to a range of SARA in the rumen fluid) and 90 mM (excessive level at ARA) according to (Counotte et al., 1981; Henning et al., 2010).

**FIGURE 4:**
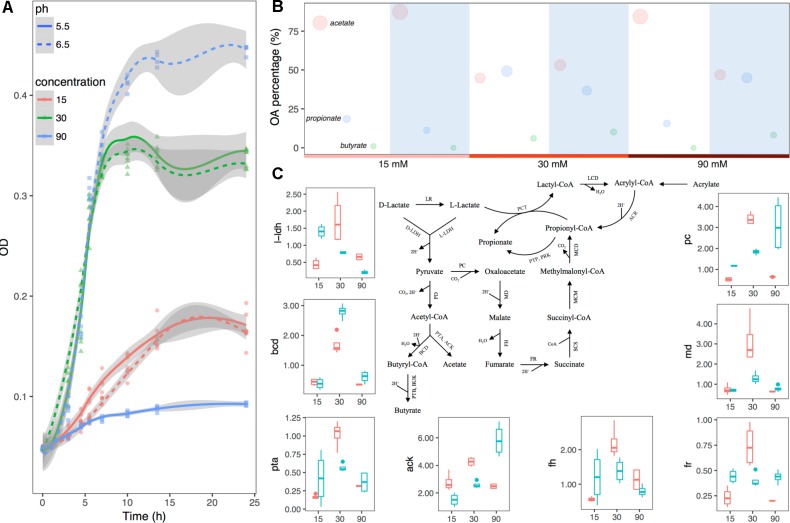
Substrate lactate abundance and pH can regulate *M. elsdenii* lactate degradation pattern in continuous culture. **(A)** Growth curves of *M. elsdenii* in medium under different conditions. **(B)** The mean proportions of acetate, propionate and butyrate in different groups. The area of the circle corresponds with the percentage of this acid. White background represents the pH of 5.5 while blue means pH of 6.5. **(C)** *M. elsdenii* lactate degradation pathways and relative expression of genes involved in metabolic pathway. Pink box means pH at 5.5 while green box represents pH at 6.6. ARC, acrylyl-CoA reductase; MCD, methylmalonyl-CoA decarboxylase; MCM, methylmalonyl-CoA mutase; SCS, succinyl-CoA synthetase (succinyl thiokinase); FR, fumarate reductase; FH, fumarase (fumarate hydratase); MD, malate dehydrogenase; PC, pyruvate carboxylase; PD, pyruvate dehydrogenase; PCT, propionyl-CoA transferase; LCD, lactyl-CoA dehydratase; LR, lactate racemase; D-LDH, D-lactate dehydrogenase; L-LDH, lactate dehydrogenase; PTP, phosphate propionyl transferase; PRK, propionate kinase; PTB, phosphate butyryl transferase; BUK, butyrate kinase; PTA, phosphate acetyl transferase; ACK, acetate kinase; BCD, butyryl-CoA dehydrogenase.

Growth curves of the bacteria with incubation time are shown in Figure 4A. Maximal growth rate at the log phase was seen in a group with pH 6.5 and lactate concentration of 90 mM, while minimal growth rate was seen in a group with pH 5.5 and lactate concentration of 90 mM (Supplementary Table S4). In order to evaluate the effects of both pH and substrate lactate doses on *M. elsdenii* lactate degradation pattern shifts, fermentation fluid was collected at 4.5 h (during the lag phase), 7 h (during the exponential phase), and 24 h (during the plateau phase) for analysis of acetate, propionate, and butyrate. Overall, with the increase of substrate lactate level, the average proportion of acetate decreased whereas propionate and butyrate increased except for in the minor growth group (Supplementary Table S5). The pH differences in different groups also had a significant impact on lactate degradation and OA production (Supplementary Table S5 and Figure 4B). Given that both substrate lactate level and pH can affect *M. elsdenii* lactate degradation, it is helpful to compare the relative importance of pH and substrate level in terms of bacteria lactate utilization characteristics. If pH plays a dominant role, the supplement of pH regulators e.g., NaHCO_3_, Al(OH)_3_ would provide good strategies to help *M. elsdenii* degrade lactate, otherwise dietary and antibacterial means should be taken to inhibit lactate production. In this case, pH actually played a more profound effect than lactate level in terms of OA production, evidenced by its greater regression coefficient values (absolute values in Supplementary Table S6) of pH compared to the values for substrate concentration.

In order to attain a better understanding of the regulator mechanism of how pH and substrate level can affect *M. elsdenii* lactate utilization, we summarized the metabolic pathway of lactate in *M. elsdenii* in Figure 4C (Hino and Kuroda, 1993; Prabhu et al., 2012; Weimer and Moen, 2013). Although *M. elsdenii’s* draft genome is sequenced (Bag et al., 2017), the genes encoding enzymes involved in lactate degradation pathways are poorly understood. Here, eight genes with annotations were selected to detect their relative expression abundance in different groups (Supplementary Table S2 and Figure 4C). The expression of *l-ldh* at a dose of 30 mM lactate was significantly higher than the lactate levels of 15 and 90 mM (*P* < 0.001), and the average concentration of total OA was also higher than the other two concentrations (Supplementary Table S5). The proportion of butyrate consisted with the expression of *bcd* in different pH and substrate level groups (Figure 4C and Supplementary Table S5). Genes involved in the propionyl-CoA pathway to produce propionate (i.e., *pc*, *fr*, *md*, and *fh*) showed a similar trend with propionate production in terms of substrate dose changes, which differed from pH changes (Figure 4C and Supplementary Table S5). Genes were also involved in the acetate pathway i.e., *ack* and *pta*, and their abundance did not correspond to acetate production (Figure 4C and Supplementary Table S5).

## Discussion

The definition of RA is primarily based on low rumen pH, typically generated in high-grain diets(Coe et al., 1999; Enemark et al., 2004; Gozho et al., 2005; Khafipour et al., 2009; Humer et al., 2015). Our *in vitro* mixed culture RA models showed great similarity with *in vivo* definitions of RA (Penner et al., 2007; Zebeli et al., 2008) in terms of pH characteristics. As a consequence of RA, the LPS level may increase in the rumen (Khafipour et al., 2009). *In vivo* study showed that LPS levels were about 700 and 7,000 ng/mL when dairy cows were fed with a low or high concentrate diet, respectively (Emmanuel et al., 2008). Here, the LPS concentrations were between 8 and 13 ng/mL, suggesting considerable difference between *in vitro* and *in vivo* models in terms of LPS concentration. Significant difference in LPS levels between *in vivo* study and present results also remained (Emmanuel et al., 2008), although the LPS level was much higher in ARA compared with normal and SARA. It was widely believed that pH-intolerant gram-negative bacterium was broken down to release LPS during cellular death and lysis (Chen and Wang, 2016; Loor et al., 2016; Plaizier et al., 2016). Mao et al. (2016) found five taxa that are positively correlated with LPS in high grain- (50% grain) induced RA in a multi-omics analysis. The high LPS levels in ARA may due to the lower pH and relative higher abundance of gram-negative *M. elsdenii* and *S. ruminantium* compare with other two models (Supplementary Figure S4). The results above support the notion that a mixed culture with different substrate starch abundances can induce similar *in vivo* RA models, but the differences still remains when compared with real *in vivo* RA.

Recent studies have demonstrated that RA is mainly induced by the accumulation of lactate in ruminants fed with a high-concentrate diet(Mao et al., 2016; Shen et al., 2017), and a dynamic mechanistic model of lactate metabolism in the rumen showed considerable consistency between lactate production and pH decline when ruminants were fed with a diet high in starch (Mills et al., 2014). MLR analysis in the present study also showed the strong effect of lactate in contributing to pH changes in terms of the total adjusted R square explained by lactate, for the first time in mixed culture RAMs (Figure 1D). Moreover, the increase of pH accompanied with the decrease of lactate in SARA model (Figure 1B, 2A) also gave reasonable interpretations of the salient role of lactate in pH changes.

The accumulation of lactate primarily depends on the balance between lactate producers and utilizers (Russell and Hino, 1985; Chen et al., 2016c; Chen and Wang, 2016). Limited lactate has been detected in SARA (Mao et al., 2016; Pan et al., 2016) due to the active role of lactate utilizers, especially *M. elsdenii* (Hernández et al., 2014; Chen et al., 2016c; Chen and Wang, 2016), and previous studies have suggested that *M. elsdenii* utilizes lactate and coverts to butyrate majorly (Long et al., 2014, 2016; Meissner et al., 2014; Muya et al., 2015; Weimer et al., 2015; Leeuw et al., 2016). For a deep understanding of OA fermentation pattern shifts at the microbial level, we explored bacteria and OA changes. Interestingly, in the SARA model, when the butyrate level increased, the relative abundance of *M. elsdenii* increased at the same time, followed by the decline of the lactate concentration, suggesting the importance of *M. elsdenii* in lactate utilization and butyrate production. Our MLR additionally considered bacteria interactions and showed the significant role of lactate utilizers, *M. elsdenii*, in interacting (competing) in lactate utilization. However, in exploring the association between bacteria and butyrate in the dynamics level, only *B. fibrisolvens* was found to be significantly associated with the butyrate production rate in the SARA model (Figure 3B). In present co-culture models, it was difficult to ascertain the real contribution of bacteria to butyrate production. Other continuous culture models have investigated the *M. elsdenii* lactate degradation under RA conditions and have demonstrated that in the SARA model (pH 5.5 and substrate lactate 30 mM) the production of both butyrate and propionate was significantly increased (Figure 4B and Supplementary Table S5). Moreover, we found that both substrate lactate and pH can regulate *M. elsdenii* lactate utilization due to the regulation of genes involved in lactate degradation, and the lactate utilization in *M. elsdenii* was more sensitive to pH changes than to substrate lactate level (Figure 4 and Supplementary Table S5). This suggests that increasing the rumen pH might provide an effective solution to help *M. elsdenii* degrade lactate in RA.

Once the competition between lactate producers and utilizers had broken down, lactate increased and the pH rapidly dropped. Given the importance of lactate in ARA, most studies focus on lactate production in ARA and rarely explore other VFA (Henning et al., 2010). A study in 2014 showed that acetate and propionate also increased significantly in a sheep ARA model (Minuti et al., 2014). However, no analysis was undertaken regarding their relationships with bacteria. In addition to the growth of *S. bovis* and *L. fermentum* that resulted in lactate accumulation in ARA, we also identified increased growth rates in *B. fibrisolvens* (in particular) and *L. fermentum*, which contributed very positively to the propionate, butyrate, and formate production rate, thus supplying us with new information regarding OA fermentation in ARA. We also noticed that some studies showed that *B. fibrisolvens* decreased in the latter period of RA and that this was not acid tolerance (Klieve et al., 2003; Khafipour et al., 2009; Fernando et al., 2010). However, here in ARA it acted as a superior flora, and contributed considerably to OA fermentation in ARA, but the cause remains unknown.

We further investigated the association between bacteria and lactate enzymes. When starch is fermented by amylolytic bacteria, it is saccharified by hydrolysis using enzymes such as α-AMY before entering into glycolysis, where it is converted into pyruvate for further catabolism in OA fermentation (Narita et al., 2004). The amount of pyruvate formed in glycolysis partly depends on α-AMY activity, and the formation of lactate is determined by LDH activity (Asanuma and Hino, 2000). Here, the average populations of amylolytic lactate bacteria and the activities of α-AMY and LDH were all significantly higher in RA models than in the normal model. At the same time, a positive association was noted among lactate producers, lactate production, and the activity of enzymes involved in lactate production both in statics and dynamics data. As a result, the average concentrations of lactate in the RA models were significantly higher than normal. Moreover, *S. bovis* contributed more in lactate production compared with *L. fermentum*, especially in the ARA model.

## Conclusion

Our results have shown that both the *in vivo* similar ARA and SARA models can be built by mixing culture of five key RA-associated anaerobic bacteria *in vitro*. The positive growth of *M. elsdenii* and *B. fibrisolvens* associated with OA accumulation in the SARA model to shift from lactate to butyrate and resulted in pH recovery. Furthermore, both the concentration of substrate lactate and pH had remarkable effects on *M. elsdenii* lactate utilization, and this may due to the transcriptional regulation of metabolic genes, and the lactate utilization in *M. elsdenii* was more sensitive to pH changes than to the substrate lactate level. In addition, compared with associations based on statics data, associations discovered from dynamics data showed greater significance and gave additional explanations regarding the relationships between bacterial competition and OA accumulation. These findings provide novel information about bacterial competition and OA fermentation pattern shifts in modeling level, enhancing our understanding of ruminal microbial ecology. But, a mixed culture of several RA associated bacteria species is far away from the real RA case, further *in vivo* studies still needed.

## Author Contributions

LC designed and conducted the experiments, analyses, and wrote the manuscript. YS, CW, LD, and FZ helped to collect the samples. HW, JF, and MW directed in experiments design and wrote the manuscript.

## Conflict of Interest Statement

The authors declare that the research was conducted in the absence of any commercial or financial relationships that could be construed as a potential conflict of interest.

## Footnotes

1 https://www.r-project.org
