# Supplementary material for: Megasphaera elsdenii Lactate Degradation Pattern Shifts in Rumen Acidosis Models
Source: Front Microbiol. 2019 Feb 7;10:162. doi: 10.3389/fmicb.2019.00162 (PMC6374331; doi:10.3389/fmicb.2019.00162)
Supplement: Supplementary file 1 [file Data_Sheet_1.docx]

Fig S1 PCR products of bacteria 16S rRNA. The left line is a DNA ladder marker (bp).


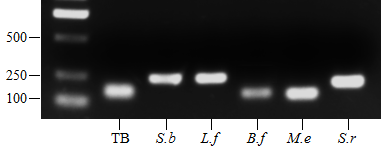


Fig S2 PCR products of genes involved in lactate metabolism pathways. The right line is a DNA ladder marker (bp).


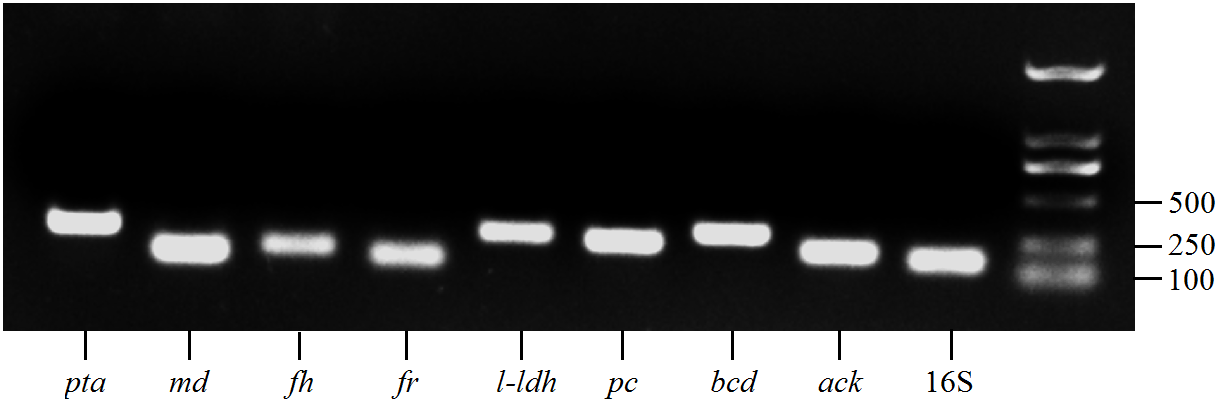


Fig S3 Proportion of bacteria (based on relative abundance) and organic acids (based on concentrations) in mixed culture models at different sampling time.


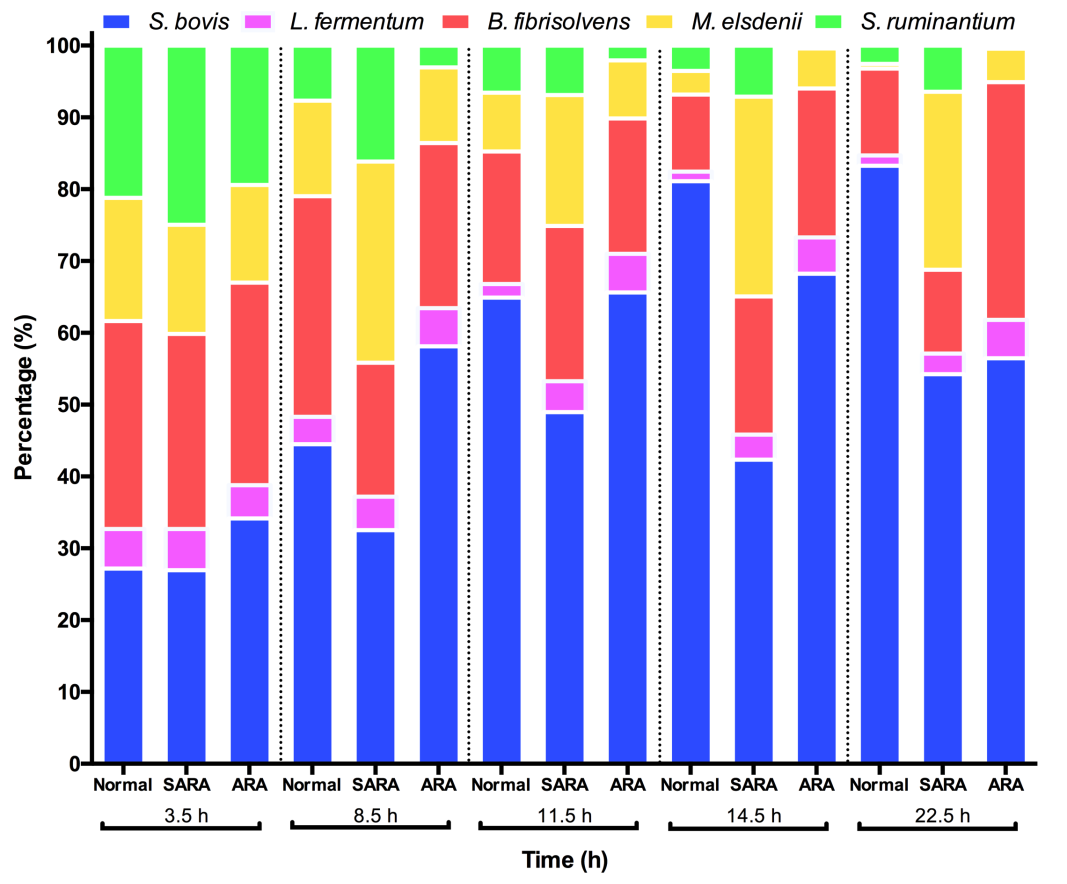

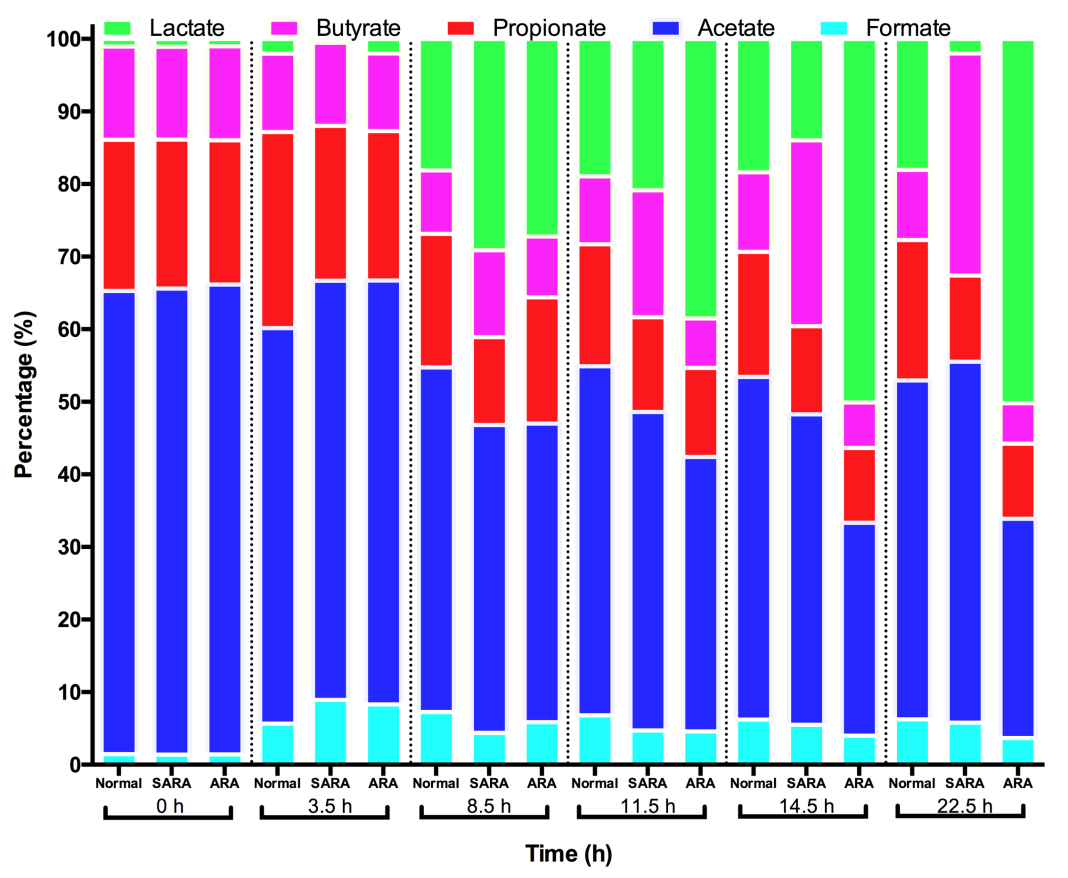


A.

B.

Fig S4 Relative abundance of bacteria (upper) and organic acids concentrations (down, m*M*) at different sampling time (hours) in different mixed culture models.

Fig S5 Activity of enzymes at different sampling time in different mixed culture models.

Fig S6 Relative growth rates of bacteria (upper) and organic acids production rates (down) at different periods in mixed culture models.
