# Supplementary material for: Megasphaera elsdenii Lactate Degradation Pattern Shifts in Rumen Acidosis Models
Source: Front Microbiol. 2019 Feb 7;10:162. doi: 10.3389/fmicb.2019.00162 (PMC6374331; doi:10.3389/fmicb.2019.00162)
Supplement: Supplementary file 2 [file Data_Sheet_2.docx]

Table S1 Oligonucleotide primers used for bacteria quantitation.

| Items | Sequence of primers (5’-3’) | Reference | Productsize (bp) | Amplification efficiency |
| --- | --- | --- | --- | --- |
| Total bacteria | F:CGGCAACGAGCGCAACCC | AbuGhazaleh et al. 2011 | 130 | 92.78% |
|  | R:CCATTGTAGCACGTGTGTAGCC |  |  |  |
| *S. bovis* | F:CGATACATAGCCGACCTGAG | [Wang et al. 2015](http://www.publish.csiro.au/?paper=AN14523) | 235 | 99.75% |
|  | R:TAGTTAGCCGTCCCTTTCTG |  |  |  |
| *L. fermentum* | F:AGCGAACAGGATTAGATACCC | [Wang et al. 2015](http://www.publish.csiro.au/?paper=AN14523) | 233 | 97.52% |
|  | R:GATGGCACTAGATGTCAAGACC |  |  |  |
| *B. fibrisolvens* | F:TAACATGAGTTTGATCCTGGCTC | [AbuGhazaleh et al. 2011](http://onlinelibrary.wiley.com/doi/10.1111/j.1439-0396.2010.01056.x/full) | 136 | 90.12% |
|  | R:CGTTACTCACCCGTCCGC |  |  |  |
| *M. elsdenii* | F:GACCGAAACTGCGATGCTAGA | [Wang et al. 2015](http://www.publish.csiro.au/?paper=AN14523) | 129 | 98.97% |
|  | R:CGCCTCAGCGTCAGTTGTC |  |  |  |
| *S. ruminantium* | F:GAGCGAACAGGATTAGATACCC | [Wang et al. 2015](http://www.publish.csiro.au/?paper=AN14523) | 194 | 96.51% |
|  | R:TGCGTCGAATTAAACCACATAC |  |  |  |

Table S2 Oligonucleotide primers used for genes involved in lactate metabolism pathways.

| Items | Sequence of primers (5’-3’) | Reference GeneBank ID | Production | Amplification efficiency |
| --- | --- | --- | --- | --- |
| 16S | F:GCCTTGACATTGATTGCTAT | NR_029207.1 | 156 | 93.17% |
|  | R:ACTTAACTCGCTGGTAACA |  |  |  |
| ack | F:TGATGCCGATGATGTTAGG | KGI89226.1 | 184 | 94.15% |
|  | R:CTCACCGATGCTGAATGT |  |  |  |
| bcd | F:CCGTTGGTGTTGAAGACT | WP_014015428.1 | 268 | 90.26% |
|  | R:CCTGCGAAGAAGTTGCTA |  |  |  |
| pc | F:GCAGTTCCAAGGCTTCAA | WP_014014983.1 | 218 | 89.74% |
|  | R:GACCAACATCGGCTTCTT |  |  |  |
| l-ldh | F:TCCAGGAACAGACCATCA | WP_014016246.1 | 239 | 95.04% |
|  | R:GTCAGGCAAGTTGTATTCG |  |  |  |
| fr | F:GCTTCGCCGTTCTTATTG | KGI90321.1 | 115 | 98.26% |
|  | R:ATGCCATCCAGTATCATCC |  |  |  |
| fh | F:TTGTCATAACCGACGAGAG | KGI88905.1 | 157 | 94.23% |
|  | R:ACCTGATCCAGTCCATCC |  |  |  |
| md | F:CCACGACGACCTGAGTAT | KGI89128.1 | 122 | 88.77% |
|  | R:CGGATACGACAGCAATCAT |  |  |  |
| pta | F:AATATCGGATGCTGGAAGAT | KGI89705.1 | 242 | 90.08% |
|  | R:GGAACTGCTGACCGTATC |  |  |  |

Table S3 Differences among co-culture models in bacteria growth and fermentation based on two-way ANOVA. Substrate starch concentrations are 1 g/liter (Normal), 3 g/liter (SARA), and 9 g/liter (ARA). A, b and c means in the same row with different superscripts differ significantly for treatment effect.

| Items | Model | | | SEM_Total_ | *P*-value | | |
| --- | --- | --- | --- | --- | --- | --- | --- |
|  | Normal | SARA | ARA |  | *P*_C_ | *P*_time_ | *P*_C×time_ |
| OD_600_ | 0.13c | 0.38a | 0.33b | 0.003 | <0.001 | <0.001 | <0.001 |
| Total sugar (g/L) | 0.47c | 0.97b | 5.94a | 0.012 | <0.001 | <0.001 | <0.001 |
| pH | 6.33a | 6.26b | 5.77c | 0.005 | <0.001 | <0.001 | <0.001 |
| *S. bovis* | 3.05c | 4.19b | 9.40a | 0.076 | <0.001 | <0.001 | <0.001 |
| *L. fermentum* | 0.14c | 0.38b | 0.82a | 0.012 | <0.001 | <0.001 | <0.001 |
| *B. fibrisolvens* | 1.01c | 1.79b | 4.04a | 0.036 | <0.001 | <0.001 | <0.001 |
| *M. elsdenii* | 0.41c | 2.31a | 1.12b | 0.032 | <0.001 | <0.001 | <0.001 |
| *S. ruminantium* | 0.42b | 0.99a | 0.41b | 0.017 | <0.001 | <0.001 | <0.001 |
| Lactate (m*M*) | 9.34b | 8.91b | 32.72a | 0.201 | <0.001 | <0.001 | <0.001 |
| Formate (m*M*) | 3.98b | 3.70c | 4.28a | 0.037 | <0.001 | <0.001 | <0.001 |
| Acetate (m*M*) | 34.82b | 35.15b | 37.25a | 0.191 | <0.001 | <0.001 | <0.001 |
| Propionate (m*M*) | 13.59a | 10.48b | 12.30a | 0.119 | <0.001 | <0.001 | <0.001 |
| Butyrate (m*M*) | 7.08b | 13.57a | 7.24b | 0.079 | <0.001 | <0.001 | <0.001 |
| α-AMY (U/L) | 8.65c | 18.53a | 11.33b | 0.202 | <0.001 | <0.001 | <0.001 |
| LDH (U/L) | 6.97c | 8.63b | 10.78a | 0.090 | <0.001 | <0.001 | 0.002 |
| LPS (ng/L) | 8.81b | 8.94b | 9.52a | 0.058 | <0.001 | <0.001 | <0.001 |

Table S4 Linear regression analysis of the growth rate of *M. elsdenii* during log phase in continuous culture.

| pH | Concentration (m*M*) | *k*^a^ | SEM*_k_* | R^2^_adjust_ | *P_R_* |
| --- | --- | --- | --- | --- | --- |
| 6.5 | 15 | 0.009 | 0.001 | 0.897 | < 0.001 |
|  | 30 | 0.052 | 0.003 | 0.959 | < 0.001 |
|  | 90 | 0.070 | 0.005 | 0.925 | < 0.001 |
| 5.5 | 15 | 0.013 | 0.001 | 0.855 | < 0.001 |
|  | 30 | 0.063 | 0.005 | 0.905 | < 0.001 |
|  | 90 | 0.005 | 0.001 | 0.817 | < 0.001 |

^a^ The slope of the growth curve during log phase.

Table S5 Differences among continuous culture models in *M. elsdenii* lactate metabolism based on three-way ANOVA. ^a, b, c^ Means different superscripts differ significantly (P < 0.05)

| Items | pH | Concentration (m*M*) | | | | Mean_pH_ | | SEM | | *P*-value | | | | | | | |
| --- | --- | --- | --- | --- | --- | --- | --- | --- | --- | --- | --- | --- | --- | --- | --- | --- | --- |
|  |  | 15 | 30 | 90 |  | |  | | *P*_pH_ | | *P*_C_ | *P*_Time_ | *P*_pH×C_ | *P*_pH×Time_ | *P*_C×Time_ | *P*_pH×C×Time_ |  |
| Lactate  (m*M*) | 6.5 | 11.43 | 10.35 | 36.51 | 19.43^b^ | | 0.159 | | <0.001 | | <0.001 | <0.001 | <0.001 | <0.001 | <0.001 | <0.001 |  |
|  | 5.5 | 9.53 | 8.01 | 80.39 | 32.64^a^ | |  |  |  |  |  |  |  |  |  |  |  |
|  | Mean_C_ | 10.48^b^ | 9.18^c^ | 58.45^a^ |  | |  |  |  |  |  |  |  |  |  |  |  |
| Acetate  (m*M*) | 6.5 | 11.66 | 13.51 | 17.63 | 14.27^a^ | | 0.130 | | <0.001 | | <0.001 | <0.001 | <0.001 | <0.001 | <0.001 | <0.001 |  |
|  | 5.5 | 14.10 | 12.60 | 11.34 | 12.68^b^ | |  |  |  |  |  |  |  |  |  |  |  |
|  | Mean_C_ | 12.88^b^ | 13.06^b^ | 14.49^a^ |  | |  |  |  |  |  |  |  |  |  |  |  |
| Propionate  (m*M*) | 6.5 | 1.45 | 8.59 | 14.95 | 8.33^a^ | | 0.070 | | <0.001 | | <0.001 | <0.001 | <0.001 | <0.001 | <0.001 | <0.001 |  |
|  | 5.5 | 3.33 | 12.20 | 2.10 | 5.87^b^ | |  |  |  |  |  |  |  |  |  |  |  |
|  | Mean_C_ | 2.39^c^ | 10.39^a^ | 8.52^b^ |  | |  |  |  |  |  |  |  |  |  |  |  |
| Butyrate  (m*M*) | 6.5 | 0.00 | 2.70 | 4.02 | 2.24^a^ | | 0.039 | | <0.001 | | <0.001 | <0.001 | <0.001 | <0.001 | <0.001 | <0.001 |  |
|  | 5.5 | 0.20 | 1.96 | 0.00 | 0.72^b^ | |  |  |  |  |  |  |  |  |  |  |  |
|  | Mean_C_ | 0.10^c^ | 2.33^a^ | 2.01^b^ |  | |  |  |  |  |  |  |  |  |  |  |  |
| Total OA  (m*M*) | 6.5 | 13.12 | 24.80 | 36.60 | 24.84^a^ | | 0.161 | | <0.001 | | <0.001 | <0.001 | <0.001 | <0.001 | <0.001 | <0.001 |  |
|  | 5.5 | 17.62 | 26.75 | 13.44 | 19.27^b^ | |  |  |  |  |  |  |  |  |  |  |  |
|  | Mean_C_ | 15.37^b^ | 25.78^a^ | 25.02^a^ |  | |  |  |  |  |  |  |  |  |  |  |  |
| Acetate  (%) | 6.5 | 88.80 | 53.13 | 46.91 | 62.95^b^ | | 0.232 | | <0.001 | | <0.001 | <0.001 | <0.001 | <0.001 | <0.001 | <0.001 |  |
|  | 5.5 | 80.43 | 44.72 | 84.40 | 69.85^a^ | |  |  |  |  |  |  |  |  |  |  |  |
|  | Mean_C_ | 84.62^a^ | 48.93^c^ | 65.66^b^ |  | |  |  |  |  |  |  |  |  |  |  |  |
| Propionate  (%) | 6.5 | 11.20 | 36.75 | 44.94 | 30.96^a^ | | 0.236 | | <0.001 | | <0.001 | <0.001 | <0.001 | <0.001 | <0.001 | <0.001 |  |
|  | 5.5 | 18.54 | 49.20 | 15.60 | 27.78^b^ | |  |  |  |  |  |  |  |  |  |  |  |
|  | Mean_C_ | 14.87^c^ | 42.98^a^ | 30.27^b^ |  | |  |  |  |  |  |  |  |  |  |  |  |
| Butyrate  (%) | 6.5 | 0.00 | 10.12 | 8.16 | 6.09^a^ | | 0.112 | | <0.001 | | <0.001 | <0.001 | <0.001 | <0.001 | <0.001 | <0.001 |  |
|  | 5.5 | 1.03 | 6.08 | 0.00 | 2.37^b^ | |  |  |  |  |  |  |  |  |  |  |  |
|  | MeanC | 0.52^c^ | 8.10^a^ | 4.08^b^ |  | |  |  |  |  |  |  |  |  |  |  |  |

Table S6 Linear regression of lactate metabolism in *M. elsdenii*.

| Items | Variable | R^2^_adjust_ | Coefficient | SME*_b_* | *P-*value | |
| --- | --- | --- | --- | --- | --- | --- |
|  |  |  |  |  | *P*_R2_ | *P_b_* |
| Lactate | pH | 0.727 | -0.451 | 0.126 | <0.001 | 0.001 |
|  | C |  | 0.024 | 0.77 |  | <0.001 |
|  | Time |  | -0.033 | -0.287 |  | <0.001 |
| Acetate | pH | 0.306 | 0.31 | 0.196 | <0.001 | 0.119 |
|  | C |  | 0.004 | 0.003 |  | 0.16 |
|  | Time |  | 0.062 | 0.011 |  | <0.001 |
| Propionate | pH | 0.121 | 0.431 | 0.221 | 0.008 | 0.055 |
|  | C |  | 0.009 | 0.003 |  | 0.013 |
|  | Time |  | 0.02 | 0.013 |  | 0.12 |
| Butyrate | pH | 0.229 | 0.63 | 0.207 | <0.001 | 0.003 |
|  | C |  | 0.007 | 0.003 |  | 0.034 |
|  | Time |  | 0.038 | 0.012 |  | 0.002 |
